# Bacterial Pneumonia and Cryptogenic Pleuritis after Probable Monkeypox Virus Infection: A Case Report

**DOI:** 10.3390/idr15060071

**Published:** 2023-12-13

**Authors:** Hubert Dawid Ciepłucha, Mateusz Bożejko, Paweł Piesiak, Sylwia Serafińska, Bartosz Szetela

**Affiliations:** 1 Department of Infectious Diseases, Liver Diseases and Acquired Immune Deficiencies, Wroclaw Medical University, 51-149 Wroclaw, Poland; 2 Faculty of Medicine, Wroclaw Medical University, 50-367 Wroclaw, Poland; 3 Lower Silesian Oncology, Pulmonology and Hematology Center, 53-439 Wroclaw, Poland; 4 Department of Infectious Diseases and Hepatology, Wroclaw Medical University, 51-149 Wroclaw, Poland

**Keywords:** STI, MPXV, pleuritis, pneumonia

## Abstract

A large number of monkeypox (MPOX) cases have been reported in Europe and North America in 2022, and a new outbreak of this disease was declared. We describe a case of a patient with probable monkeypox during the height of this epidemic in Poland. The patient’s symptoms resolved within two weeks, but over the next two months, he developed community-acquired pneumonia requiring hospitalization and, subsequently, non-specific pleuritis. The simultaneous occurrence of such severe infections in a previously healthy young man is not typical and suggests a potential underlying cause. We believe the potential association of these diseases with probable monkeypox virus infection is very likely. Cases of monkeypox pneumonia, both viral and secondary bacterial, have already been reported in the literature. Cases of viral pleuritis in the course of MPOX in animals have also been described; however, to our knowledge, no similar cases have been described in humans to date. Our case indicates that it is important to monitor patients after MPOX in order to respond promptly to potentially life-threatening but, as of yet, not fully understood complications.

## 1. Introduction

Monkeypox (MPOX) was first described in 1958 in two groups of primates in research farms where two smallpox-like outbreaks occurred. The first human cases were reported in 1970 in what is now the Democratic Republic of the Congo [1]. Infection with monkeypox virus (MPXV) has been reported sporadically in humans, with the vast majority of cases until 2003 in central Africa [2]. Subsequent cases were reported in 2017–2018 in Nigeria, 2018 in Great Britain, and 2019 in Singapore. In 2022, a large number of cases were registered in Portugal, Spain, the USA, and Sweden, and a new outbreak was declared [3]. The first Polish case was reported on 10 June 2022 [4].

We present a case of probable MPXV infection in a 34-year-old man who gave informed consent to publish a description of his case. Due to organizational reasons, virological confirmation of the diagnosis was not possible. The differential diagnosis should also consider varicella–zoster virus, herpes simplex virus, enteroviruses and molluscum contagiosum virus (which cause common human infections), as well as vaccinia virus (contained in smallpox vaccines), cowpox virus and paravaccinia virus (causing cowpox and pseudocowpox, respectively, which are zoonotic diseases). However, based on clinical symptoms, history, and the epidemiological situation in Poland at the time, we believe that MPXV infection was the most likely cause, which we discuss in more detail in the Section 3. Discussion.

In the case we described, two months post-probable MPXV infection in the patient, he developed community-acquired pneumonia requiring hospitalization and, subsequently, non-specific pleuritis. In this paper, we would like to pose the question of whether there could have been a link between the initial infection and the occurrence of the aforementioned pulmonary disorders in the patient. Pleuritis caused by MPXV infection has only been reported in animals, not humans [5]. However, MPXV is known to have negative effects on the human immune system, including impaired natural killer cell function, lymphopenia, increased antibody levels, increased numbers of monocytes and granulocytes in the blood, cytokine storm, complement system inhibition, and antibody-dependent enhancement [6]. Although finding a definitive answer to the question we posed about the potential link between initial infection and pulmonary disorders has not been possible at this time, we believe that the present description is of great exploratory value. It suggests the possibility of non-obvious complications following viral infection. Perhaps in the future, similar cases will be described, or other studies of the potential long-term effects of MPXV infection on human health will be conducted.

## 2. Case Report

**Figure 1:**
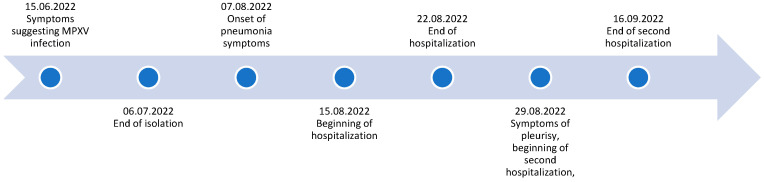
Timeline of the described clinical case.

**Figure 2:**
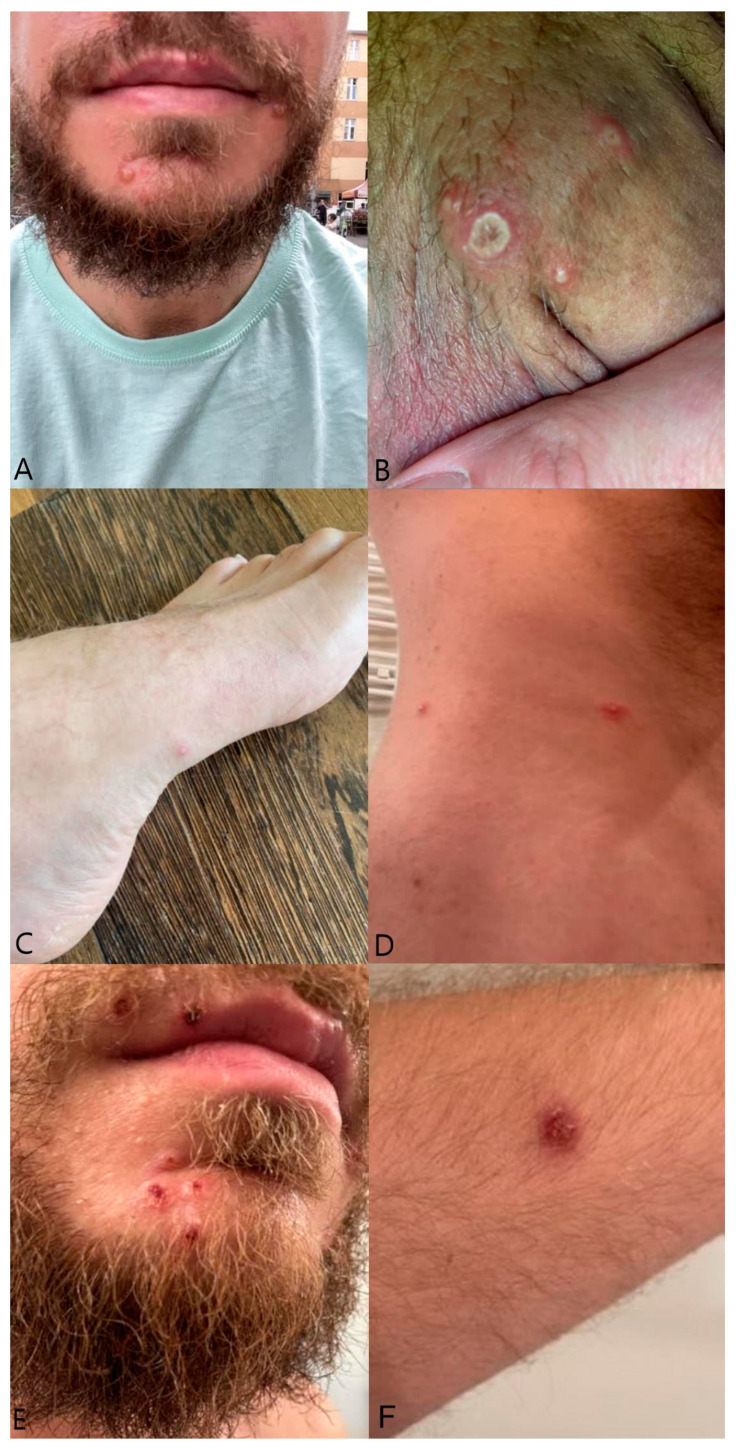
Diffuse soft large vesicles on the face and large umbilicated vesicles on the erythematous surface on the penis’ shaft seen during first physical examination (inserts (**A**,**B**)). Small disseminated vesicles seen on foot and other areas two days after primary eruptions (inserts (**C**,**D**)). Vesicle scabbing on the face and on forearm two weeks after primary eruptions (inserts (**E**,**F**)).

A 34-year-old man, identified as a member of the MSM (men having sex with men) community, with a history of syphilis and circuit party involvement, visited an outpatient clinic on 15 June 2022 after a short visit to Berlin. The timeline of case is presented in Figure 1 and general information about patient is in Table 1. He complained of malaise, fever, and sore throat and exhibited umbilicated vesicles on an erythematous surface, mainly in the genital area, with isolated vesicles present on the trunk and extremities and soft large vesicles on his face (Figure 2). He also experienced redness and tenderness of the mouth and pharynx mucosa. The previous day, the patient developed a fever of 38 degrees Celsius, malaise, muscle pain, and headache with neck, axillary, and inguinal lymphadenopathy. The patient reported that he had a regular partner but, at the same time, reported risky sexual contact with other partners and daily use of HIV pre-exposure prophylaxis (PrEP). He had not been vaccinated against smallpox. A probable diagnosis of monkeypox was made based on the epidemiological situation at the time (height of MPOX epidemics in Poland/Europe) and the characteristic clinical picture (patient belonging to the MPOX risk group, characteristic skin lesions with central and then distal dissemination, characteristic lymphadenopathy, mucosa involvement, self-limiting course with characteristic scabbing, no other pathogens confirmed). All these elements indicated that the diagnosis of monkeypox was very probable. Virological confirmation was not possible as the patient decided to rest and isolate at home. Symptoms resolved in two weeks. Despite close contact, the patient’s regular partner did not develop symptoms of the disease, which could be explained by childhood vaccination against smallpox.

On 15 August 2022, the same patient was urgently hospitalized due to suspected community-acquired pneumonia, the basic information about this hospitalization are presented in Table 2. He reported malaise, cold symptoms, and a productive cough (lasting about a week), in addition to a fever exceeding 39 degrees Celsius, hemoptysis (pink-colored sputum), chest pain, and dyspnea. Physical examination revealed moist skin, tachypnea (about 35 breaths/min), and muted alveolar murmur over the base of the left lung and crackles over the right lung. Peripheral oxygen saturation without supplemental oxygen therapy was 94%. Laboratory tests showed elevated levels of C-reactive protein (280.76 mg/L), procalcitonin (10.002 ng/mL), creatinine (1.24 mg/dL), and urea (72.5 mg/dL). The white blood cell count on the day of admission was normal at 7.45 × 10^3^/μL, with an increased percentage of neutrophils (82.8%) and decreased lymphocytes (10.5%). Sepsis was suspected, prompting empirical intravenous antibiotic therapy with ceftriaxone (2 g per day until discharge home, that is, for eight days) and vancomycin (1 g every twelve hours for eight days) was started. Ceftriaxone was used as a broad-spectrum beta-lactam antibiotic active against *Streptococcus pneumoniae* and other bacteria that are the most common causes of community-acquired pneumonia, and vancomycin as an antibiotic also active against MRSA.

**Figure 3:**
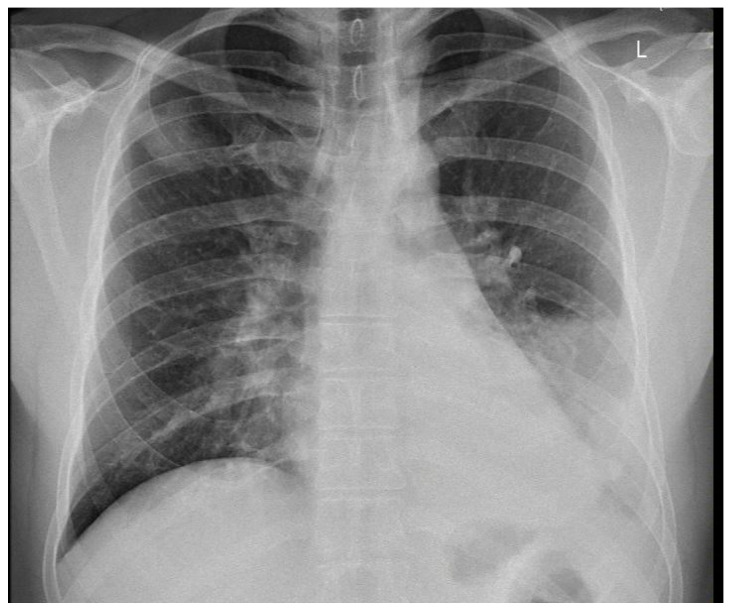
Left lung lower lobe infiltrates are shown, as well as a small amount of fluid in the left pleural cavity at X-ray scan.

**Figure 4:**
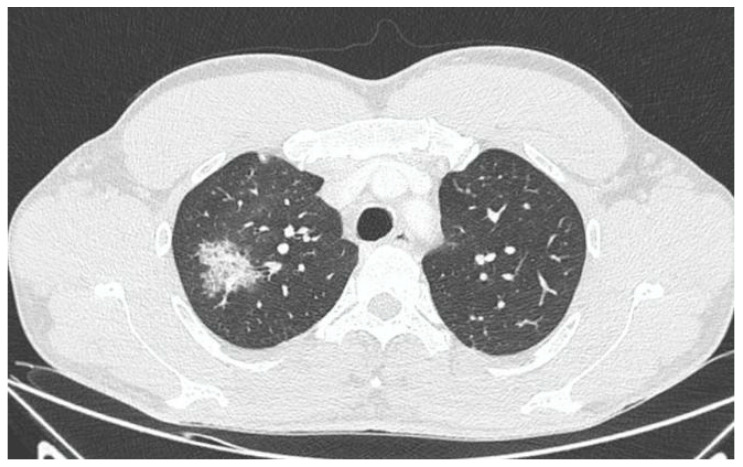
CT scan of the chest showing right lung upper lobe interstitial changes during first hospitalization.

X-ray and CT scans of the chest were performed. The left lung’s lower lobe infiltrates and the right lung’s upper lobe interstitial changes (most probably of an inflammatory nature) were shown, as well as a small amount of fluid in the left pleural cavity (Figure 3 and Figure 4). Several tests were also performed to determine the etiology of the pneumonia. However, blood cultures, an antigen test for SARS-CoV-2, urine tests for *Streptococcus pneumoniae*, *Legionella pneumophila*, *Cryptococcus* sp., and *Mycobacterium tuberculosis* antigens, and an HIV screening test were all negative. In the following days, empirical antibiotic therapy with ceftriaxone and vancomycin was continued. A rapid improvement in the patient’s clinical condition was observed, as well as a significant decrease in the levels of inflammatory markers. On 22 August 2022, the patient was discharged home in good general condition.

**Figure 5:**
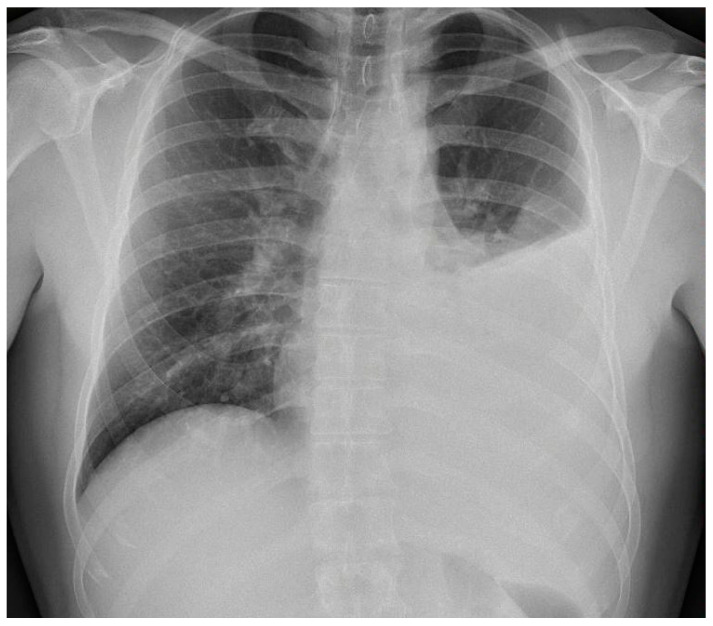
Chest X-ray during second admission to the ward revealed fluid in the left pleural cavity reaching the third rib.

On August 27th, the patient again presented to an outpatient clinic because of stabbing pain on the left side of the chest. Information about the second hospitalization is available in Table 3. Due to a dull percussion sound over the left lung, a chest X-ray was taken and revealed fluid in the left pleural cavity reaching the third rib (Figure 5). The patient was readmitted to the hospital. On admission, a slightly elevated C-reactive protein level (11.51 mg/L) and a normal procalcitonin level (0.036 ng/mL) were found. Empirical intravenous antibiotic therapy with ceftriaxone (2 g per day for 10 days) and amikacin (500 mg twice per day for 10 days; used in addition to a broad-spectrum beta-lactam because of suspected bacteremia and broad-spectrum activity against Gram-negative bacteria) was implemented.

A series of diagnostic tests was performed. Blood cultures, PCR for *Mycobacterium tuberculosis* in pleural fluid, antinuclear antibody and rheumatoid factor tests, and antigen test for SARS-CoV-2 were all negative. Thoracocentesis was performed twice, one week apart. The fluid obtained was an exudate with a predominance of polymorphonuclears over mononuclears (the first time, PMNs accounted for 55.7% and MNs 44.3%; the second time, PMNs were 65.3% and MNs were 34.7%).

Despite thoracocentesis, a further increase in fluid in the left pleural cavity and an increase in the level of C-reactive protein to 67.70 mg/L (with a simultaneous decrease in the level of procalcitonin to 0.021 ng/mL) were observed, and the treatment seemed ineffective. Therefore, on the tenth day of hospitalization, the patient was transferred to the pulmonology department. A pleural ultrasound exam revealed fluid and numerous adhesions in the pleural cavity. Percutaneous Abrams pleural biopsy was performed, and a pleural drain was inserted (six days after the last thoracocentesis). The fluid was exudate again, this time with a predominance of lymphocytes (85%) over neutrophils (7%); no malignant cells were found in the fluid. Histopathological examination of the pleural sample was inconclusive, with fragments of necrotic tissue and lymphocytic exudate with dispersed diffuse atypical cells of pan Ck (+), Ck 7 (+), Ck 20 (−), WT-1 (−), calretinin (−), TTF-1 (−), p63 (−), LCA (−), and CD68 (−) phenotypes. This histopathological picture may indicate both inflammatory and neoplastic processes. PCR, culture, and bacterioscopy for *Mycobacterium tuberculosis* in the pleural fluid, pleural specimen and bronchial lavage, pleural fluid culture, bacteriological culture and mycological examination of bronchial lavage, antigen test for *Chlamydophila pneumoniae*, HIV screening, anti-myeloperoxidase IgG, and anti-proteinase 3 IgG were all negative.

Despite the use of only symptomatic treatment (pleural drainage and analgesics), a gradual improvement in the patient’s general condition and a decrease in the level of C-reactive protein were observed. Due to the reduction in the amount of fluid in the left pleural cavity, the drain was removed. On September 16th, the patient was discharged in good condition with a planned follow-up chest CT scan and a whole-body PET-CT to exclude the neoplastic etiology of pleuritis. One month after discharge, a chest CT scan showed a minimal amount of fluid in the left pleural cavity (almost complete regression compared to the previous month) and a regression of the infiltrates in the lungs. The patient’s condition remained good, with no recurrence of symptoms. The PET-CT scan showed no active process, which might be considered malignant. The etiology of the pleuritis remains unconfirmed.

## 3. Discussion

The case is complex and involves many aspects. After a seemingly self-limited probable MPXV infection, the patient developed severe community-acquired pneumonia. The entire clinical picture and the levels of procalcitonin, creatinine, and urea at admission to the hospital suggest that the patient would have developed sepsis if appropriate antibiotic therapy had not been administered in time. Additionally, the patient developed non-specific pleuritis, the definitive cause of which remains unclear. The simultaneous occurrence of such severe infections in a previously healthy young man (without comorbidities) is atypical and warrants consideration of an additional potential cause.

The patient’s initial infection occurred during the peak of the MPOX epidemic among MSM in Poland. The patient’s history, such as recent risky sexual behavior and a recent trip to Berlin, and clinical symptoms were typical of MPOX. The appearance of the skin lesions and their typical progression were particularly characteristic. However, since the patient did not consent to hospitalization, PCR testing to confirm MPXV infection was not possible under the conditions of the Polish public health care system. Therefore, we cannot definitively state whether the patient was infected with this virus. In the differential diagnosis, the main considerations should be varicella–zoster virus (causing chickenpox and shingles), herpes simplex virus (causing genital herpes, among others), enteroviruses (causing hand, foot, and mouth disease, among others), and molluscum contagiosum virus. However, the patient had already had chickenpox in childhood, and the symptoms did not indicate shingles (in which there is, among other things, pain in the involved dermatome). A different progression of skin lesions and the presence of single lesions on the trunk and extremities argued against genital herpes. Hand, foot, and mouth disease occurs mainly in children and is characterized by skin lesions mainly in other parts of the body than in this case. The appearance of the skin lesions was also very different from those found in molluscum contagiosum. Symptoms very similar to those seen in this case can be caused by other poxviruses: vaccinia virus, cowpox virus, and paravaccinia virus. However, the patient had not been vaccinated against smallpox or had no contact with animals transmitting these viruses. Thus, based on the overall clinical picture, we believe that MPXV infection was the most likely cause, although, of course, other causes cannot be completely excluded. We classify this case as probable MPXV infection, based on the diagnostic criteria that were in force in some countries at the time of the MPXV epidemic (among others in the United Kingdom), allowing this case to be considered a probable MPXV infection on the basis of the epidemiological situation, history, and clinical symptoms.

In this paper, we posed the question of whether there is a possible link between the initial infection and the subsequent occurrence of pulmonary disorders in the patient. Monkeypox viral pneumonia is one of the more commonly described complications of this disease in humans and can be associated with significant mortality [7,8,9,10,11]. Goff et al. published the results of an animal study (on primates), which concluded that monkeypox pneumonia can be lobular or interstitial pneumonia of viral etiology or can be caused by bacterial superinfection [10]. In the case we described, pneumonia occurred after the symptoms of the initial infection had already resolved and most likely was of bacterial etiology. Although it was not possible to identify any specific pathogen, bacterial etiology is supported by the overall clinical picture and, above all, the rapid resolution of symptoms after the administration of antibiotics. However, we cannot exclude the superposition of viral and bacterial pneumonia (in particular, bacterial superinfection of a primary viral infection). This possibility may be indirectly indicated by the heterogeneous radiological picture. In X-ray and CT scans, inflammatory lesions were visible in both lungs. However, while in the left lung, they were described as lobular pneumonia, in the right lung, there was an area of likely interstitial inflammatory lesions. It should be mentioned that the literature indicates that bacterial superinfection can exacerbate the symptoms of monkeypox viral pneumonia [10,12].

The more mysterious aspect of the described case is the non-specific pleuritis; its etiology has not been identified despite a great variety of diagnostic tests. We believe that a viral etiology should be considered, as it is a common cause of non-specific pleuritis [7,8]. It should also be noted that the exudative fluid in the course of viral pleuritis may be dominated by both lymphocytes and granulocytes [9]. In the reported case, the suspicion of a viral etiology may be raised indirectly by an increase in the level of C-reactive protein (after a previous decrease during the treatment of pneumonia), with a simultaneous decrease in the level of procalcitonin, as well as the lack of response to antibiotic therapy. To the best of our knowledge, no case of pleuritis in humans has been described after MPOX to date. However, such cases have been described in animals [5,10]. In a study published by Goff et al., pleuritis occurred in six of nine monkeys exposed to MPXV [10].

Michailidou et al. described a case of pleuritis in which viral etiology (HSV-1) superimposed on bacterial pneumonia and pleuritis [11]. If the pneumonia was strictly bacterial, the pleuritis could have been of viral etiology. Among the viruses most likely to cause pleuritis (especially in immunocompromised patients) are influenza viruses, parainfluenza viruses, respiratory syncytial virus, herpes simplex virus, cytomegalovirus, measles virus, varicella–zoster virus, and adenovirus [9]. However, it cannot be completely excluded that transient immunosuppression after bacterial pneumonia may have caused the reactivation of an incompletely eradicated probable MPXV infection.

In our report, a factor that undermines a possible direct link between MPXV infection and pulmonary disorders is the relatively long time (around one month) between the probable onset of MPOX and the development of pneumonia and pleuritis. However, the results of an animal study published by Goff et al. show that primates exposed to MPXV who survived the disease had lung and pleural lesions when they were euthanized 21–25 days after the infectious challenge [10]. Citing the results of this study, Reynolds et al. conclude that possible temporary or longer-term sequelae should also be considered in people recovering from monkeypox and that observation of these patients can help determine the severity and duration of potential pulmonary complications of this disease [12]. In this context, we believe that the pulmonological disorders we have described could potentially be the unusual complications after MPOX in humans. However, it should be noted that the results of studies in animals that were infected by inhaled high doses of a more virulent strain of the virus cannot be directly related to the case described. However, these studies may, to some extent, suggest at least the possibility of a link between MPXV infection and the pulmonary disorders we described. It should also be added that although our patient was probably infected with MPOX through sexual contact, aspiration of the virus through the respiratory tract (particularly during sexual contact) cannot be excluded.

In summary, it is currently impossible to clearly determine the etiology of the pulmonary disorders that occurred in the case we described. Nevertheless, even if the pneumonia and non-specific pleuritis were not directly related to the initial infection, this infection may have indirectly contributed to these disorders. The viral infection may have caused a transient immunosuppression that predisposed the patient to develop other infections. Many viruses can cause transient immunosuppression by various mechanisms. A typical example is the measles virus, which can cause immunosuppression lasting up to 6 months [13]. Among viruses of the genus Orthopoxvirus, to which MPXV belongs, the immunosuppressive effect of the cowpox virus has been best described. The proteins it encodes have the ability to modify the host response both to the viral infection itself and to secondary infections [14,15]. As we mentioned in the introduction, immunocompromise has also been described for MPXV [6]. In addition, Hrusch et al. published the results of a study in mice, based on which they concluded that orthopoxvirus infections create an immunosuppressive microenvironment that impairs the host’s pulmonary immune responses [16]. Perhaps a similar situation occurred in the case we described.

One of the main limitations of our description is the lack of a PCR test for MPXV and other tests that could help establish or exclude an association between the described pulmonary disorders and MPXV infection. As already mentioned, it was not possible to perform these tests for organizational reasons (the patient was diagnosed and treated within the public health care system in Poland; in this system, unfortunately, the possibilities for diagnostic tests are often still very limited). It was not possible to perform PCR testing on the patient at the time of the appearance of the characteristic skin lesions because the patient did not consent to hospitalization, and outpatient performance of such tests within the public health care system is not possible in Poland. If similar cases occur in the future, performing tests that were not available in the situation we described could allow a better understanding of the observed symptoms.

## 4. Conclusions

At present, we cannot provide a definitive answer to the question of whether there is a link between the initial infection and the patient’s subsequent pulmonary disorders. We suspect that the patient probably had an MPXV infection. However, due to the impossibility of conducting PCR testing, we cannot conclusively exclude another cause for the initial infection. The patient’s pneumonia was most likely of bacterial etiology. Regarding pleuritis, despite extensive diagnostic efforts, a definitive determination of the etiology has not been possible thus far. Nevertheless, numerous factors suggest a viral etiology. Concurrently, even if the initial infection was not directly linked to the subsequent disorders, it might have induced transient immunosuppression, elevating the risk of subsequent infections, including pneumonia and pleurisy.

For future patients presenting similar symptoms, it would be highly advisable to confirm MPXV infection by PCR test, which was not possible in this case. PCR tests of pleural fluid and pleural samples for MPXV and other viruses that commonly cause pleuritis would also be advisable. To investigate the possible immunosuppressive impact of MPXV on the patient’s body warrants additional tests, such as measuring cytokine levels and analyzing lymphocyte subpopulations. Performing these tests could assist in definitively establishing or excluding an association between the described pulmonary disorders and MPXV infection.

Monitoring the clinical condition of people after MPOX is crucial to promptly address potentially life-threatening and yet unknown complications. Similarly, emphasizing the importance of monitoring patients after bacterial pleuritis is essential, considering the possibility of developing viral pleuritis after the resolution of pneumonia symptoms.

## Figures and Tables

**Table 1:** Basic information about the patient.

Age	34
Gender	Male
Co-existing diseases	None
Chronic medication	Emtricitabine/tenofovir disoproxil 200 mg/245 mg as pre-exposure prophylaxis for HIV infection
Ethnicity	Caucasian

**Table 2:** Basic information about the first hospitalization.

Date of Hospitalization	15–22 August 2022
Diagnosis	Lobar pneumonia
Symptoms	Fever of 39 degrees Celsius, productive cough, chest pain
CRP level	280.76 mg/L
Procalcitonin level	10.002 ng/mL
Creatinine level	1.24 mg/dL
Urea level	72.5 mg/dL
White blood cell level	7.45 × 10^3^/μL
Blood smear	Increased percentage of neutrophils (82.8%), decreased lymphocytes (10.5%).
IGRA test	Negative
Anti-HIV + p24	Negative
SARS-CoV-2 antigen	Negative
*S. pneumoniae* antigen in urine	Negative
*L. pneumophilia* antigen in urine	Negative
Aerobic blood cultures	Negative
Anaerobic blood culture	Negative
PCR *M. tuberculosis*	Negative
CT scan	In the lower lobe of the left lung, an area of infiltrative changes with an air bronchogram of inflammatory changes is visible. An irregular, non-homogeneous circular area of interstitial changes with a diameter of 3 cm is visible in the upper lobe of the right lung. Probably inflammatory changes. In the right apex, a second similar lesion with a diameter of 1.5 cm is visible subpleurally.

**Table 3:** Basic information about the second hospitalization.

Date of Hospitalization	29 August–16 September 2022
Diagnosis	Pleural effusion, pleurisy
Symptoms	Stabbing pain on the left side of the chest, discomfort
CRP level	11.5 mg/L
Procalcitonin level	0.036 ng/mL
Creatinine level	0.89 mg/dL
Urea level	38.6 mg/dL
White blood cell level	8.68 × 103/μL
Blood smear	Without deviations from the population norm
PCR of *M. tuberculosis* from bronchial lavage	Negative
PCR of *M. tuberculosis* from pleural effusion	Negative
PCR of *M. tuberculosis* from a pleural biopsy	Negative
Bacterioscopy for *M. tuberculosis* infection of bronchial tree lavage	Negative
Mycological examination of bronchial lavage	Negative
Aerobic/anaerobic culture of pleural effusion fluid	Negative
Aerobic/anaerobic culture of bronchial lavage fluid	Negative
Aerobic/anaerobic culture of blood	Negative
SARS-CoV-2 antigen	Negative
Antigen test for *Ch. pneumoniae*	Negative
Anti-myeloperoxidase IgG	Negative
Anti-proteinase 3 IgG	Negative
Anti-HIV + p24	Negative
Antinuclear antibody	Negative
Rheumatoid factor	Negative
Chest X-ray	The image indicates the presence of a large amount of fluid in the left pleural cavity up to the level of the anterior section of the third rib

## Data Availability

All relevant data are given within the manuscript. Sensitive personal data of patients participating in this study are not available. The Patient Base is located in the Prevention and Treatment Clinic of the Wrocław Health Center in Wrocław.
